# Predicting incident radiographic knee osteoarthritis through quantitative meniscal lesion parameters: data from the osteoarthritis initiative

**DOI:** 10.1186/s12891-024-07706-5

**Published:** 2024-08-06

**Authors:** Kaida Bo, Xiangpeng Xie, Xin Liu, Jianliang Ou, Yuanyi Zhang, Xu Wang, Shuo Yang, Wei Zhang, Lelei Zhang, Jun Chang

**Affiliations:** 1 The First Affiliated Hospital of Anhui Medical University, Anhui Public Health Clinical Center, Hefei, 230000 China; 2 School of Basic Medical Sciences, Anhui Medical University, Hefei, 230000 China; 3 Inflammation and Immune Mediated Diseases Laboratory of Anhui Province, School of Life Sciences, Anhui Medical University, Hefei, 230000 China

**Keywords:** Knee, Osteoarthritis, Meniscus, Magnetic resonance imaging, Radiography, Biomechanics, Mechanical stress

## Abstract

**Background:**

This study investigates the potential of novel meniscal parameters as predictive factors for incident radiographic knee osteoarthritis (ROA) over a span of four years, as part of the Osteoarthritis Initiative (OAI) study.

**Objectives:**

Quantitative measurements of meniscal parameters alteration could serve as predictors of OA’s occurrence and progression.

**Methods and materials:**

A nested matched case-control study design was used to select participants from OAI study. Case knees (*n* = 178) were defined as those with incident ROA (Kellgren Lawrence Grade (KLG) 0 or 1 at baseline (BL), evolving into KLG 2 or above by year 4). Control knees were matched one-to-one by sex, age and radiographic status with case knees. The mean distance from medial-to-lateral meniscal lesions [Mean(MLD)], mean value of tibial plateau width [Mean(TPW)] and the mean of the relative percentage of the medial-to-lateral meniscal lesions distance [Mean(RMLD)] were evaluated through coronal T2-weighted turbo spin echo (TSE) MRI at P-0 (visit when incident ROA was found on radiograph), P-1(one year prior to P-0) and baseline, respectively. Using the imaging data of one patient, the mechanism was investigated by finite element analysis.

**Results:**

Participants were on average 60.22 years old, predominantly female (66.7%) and overweight (mean BMI: 28.15). Mean(MLD) and Mean(RMLD) were significantly greater for incident knees compared to no incident knees at baseline, P-1 and P-0. [Mean(MLD), Mean(RMLD); (42.56–49.73) mean ± (7.70–9.52) mm SD vs. (38.14–40.78) mean ± (5.51–7.05)mm SD; (58.61–68.95) mean ± (8.52–11.40) mm SD vs. (52.52–56.35) mean ± (6.53–7.85)mm SD, respectively]. Baseline Mean(MLD) and Mean(RMLD), [Adjusted OR, 95%CI: 1.11(1.07 to 1.16) and 1.13(1.09 to 1.17), respectively], were associated with incident ROA during 4 years, However, Mean(TPW) [Adjusted OR, 95%CI: 0.98(0.94 to 1.02)] was not associated with incident ROA during 4 years. While Mean(TPW) at P-1 and P-0 was not associated with the risk of incident ROA, Mean(MLD) and Mean(RMLD) at P-1 and P-0 were significantly positively associated with the risk of incident ROA.

**Conclusions:**

The meniscal parameters alteration could be an important imaging biomarker to predict the occurrence of ROA.

**Supplementary Information:**

The online version contains supplementary material available at 10.1186/s12891-024-07706-5.

## Background

Knee osteoarthritis (OA) is a common form of arthritis, and is characterized by changes in the whole joint, including cartilage damage and loss, subchondral bone edema and sclerosis, Meniscus extrusion and degeneration, synovitis, changes in the infrapatellar fat pad and ligament injuries [1, 2]. While numerous structural abnormalities exist within the joint, meniscal lesions have garnered substantial attention due to their increasingly acknowledged role in the development of ROA [3, 4].

The medial and lateral menisci, characterized by their approximate wedge and semi-lunar shapes, serve as essential spacer structures between the corresponding femoral condyle and the tibial plateau [5, 6]. These structures benefit from region-specific innervation and vascular nourishment, thereby playing a pivotal role in load distribution, shock absorption, and overall knee joint integrity [7, 8]. Moreover, their close interaction with surrounding anatomical structures underscores their functional significance. In recent years, extensive scientific research has identified the anatomical, biomechanical, and functional importance of the meniscus within the knee joint. As an important component of the joint, it prevents the deterioration and degeneration of the articular cartilage, as well as the risk of developing knee OA. Even after meniscus injury, improving meniscus function through surgical implantation of meniscus tissue engineering stents or non-surgical means can reduce the risk of OA [9, 10]. However, factors such as meniscus wear and tear that gradually increase with age or meniscus trauma can cause meniscus damage, thus changing the knee microenvironment, causing synovial cells, adipocytes, synovial inflammation, infrapatellar fat pad releases inflammatory factors, and then synovitis, causing joint pain and fluid accumulation, and even knee OA [11–13]. In light of these findings, exploring the correlation between meniscal lesions and OA has become a focal point of contemporary research.

The use of semi-quantitative measures from magnetic resonance imaging (MRI) such as meniscal extrusion and meniscal size, derived from the Whole Organ Magnetic Resonance Imaging Score (WORMS), Boston Leeds Osteoarthritis Knee Score (BLOKS), or the MRI Osteoarthritis Knee Score (MOAKS) have been suggested as significant indicators of disease progression over time [14, 15]. However, predictive efficacy of these semi-quantitative metrics has been observed to be relatively modest, with some researchers positing that these scores fail to anticipate the onset of ROA. A study conducted by Sharma et al. examined pre-radiographic MRI lesions in individuals with a stronger risk of OA but who had Kellgren Lawrence grading (KLG) 0 knees at the time of examination. Their findings indicated that meniscal extrusion, evaluated using semi-quantitative methodologies, was slightly associated (odds ratio [OR] 1.72, 95% confidence interval [CI] 0.63, 4.71), albeit non-significantly, and was present in only 14% of the knees analyzed [2]. One such study, conducted by Emmanuel et al., aimed to predict ROA via measurements of meniscus extrusion, with researchers gauging meniscal extrusion in the central five-layer section. Unfortunately, they found that the meniscal extrusion was not significant in patients with new-onset KOA (at year 3 or 4 follow-up (late incidence)) [16]. This consensus, the importance of the meniscus in OA, prompted our exploration into amalgamating multiple risk factors implicated in ROA onset into a single representative predictor, potentially enhancing our ability to predict this condition. Consequently, we introduced comprehensive parameters for both the medial and lateral meniscus, reflecting the combined influence of meniscal tears, degeneration, extrusion and other chronic factors. The new parameters proposed by us are mainly the combination of the meniscus extrusion distance and the meniscus degeneration signal, which mainly includes the mean distance between lesions on the medial and lateral menisci(Mean(MLD)), the mean value of the tibial plateau (Mean(TPW)) and mean relative lesion distance (Mean(RMLD)). These parameters indicate normal or abnormal changes in the meniscus. Measuring these parameters may help diagnose or predict the occurrence of knee OA. Our research was driven by the hypothesis that quantitative measurements of meniscal parameters alteration could serve as predictors of OA’s occurrence and progression.

This nested case-control study incorporated participants from the Osteoarthritis Initiative (OAI) study, identified as being at high risk of developing symptomatic knee ROA. We measured a number of parameters: mean (MLD), mean (TPW), and mean (RMLD) on the MRI of these included cases, however the predictive validity of these measures remains uncertain. The aim of the current nested case-control study was to determine whether the parameters of meniscus lesions were associated with an increased risk of incident ROA over 4 years in the OAI study, and we also explored pathogenesis by means of finite element analysis.

## Methods

### Study design and patients

This analysis utilized a subsample from the OAI study, a multicenter, longitudinal, retrospective data-extraction epidemiological study primarily centered on knee OA. The OAI study enrolled 4,796 participants (ages 45–79) between February 2004 and May 2006, from four distinct clinical sites within the United States. These participants were monitored for a period of four years, with annual clinical evaluations and radiological assessments (x-ray and magnetic resonance imaging). Appropriate candidates were allocated to the progression, incidence, and reference control groups. Exclusion criteria encompassed individuals with a contraindication to MRI and those diagnosed with rheumatoid arthritis or other inflammatory arthritic conditions. The specifics of subject inclusion and exclusion have been delineated in a previous publication [17]. The data examined in the present study derived from the OA incidence subcohort, comprising participants with risk factors predisposing them toward the development of symptomatic knee OA. In the study of the mechanical mechanism, we included a healthy male (BMI: 20.02 kg/m2) from our hospital as the study object.

Demographic information (age, gender, ethnicity, height and weight) was already recorded at the first visit. BMI (weight/ height ^2^ kg/m^2^ ) was calculated at the first visit. Informed consent documentation and study protocols were approved by institutional review boards of each center participating in the Osteoarthritis Initiative and the clinical management committee of the Anhui Public Health Clinical Center. The Declaration of Helsinki was followed for all experiments.

### Sample description

Case knees (*n* = 178) were identified as those belonging to participants who, at baseline, showed no ROA (KLG 0 or 1) but developed incident ROA (KLG ≥ 2) at any follow-up time point (12, 24, 36, or 48 months). This sample contained knee images at each follow-up time point. In situations where a participant developed ROA in both knees, each knee was included in the analysis. Control knees were matched one-to-one by gender, age (± 5 years) and radiographic status (KLG = 0 or 1 in the index knee) with 178 case knees. Control knees maintained their non-ROA status from BL (KLG 0 or 1) to the 48-month follow-up (KLG < 2). The initial occurrence of ROA was designated as P-0, with P-1 representing the year prior to P-0, and the baseline denoting the time of enrollment. Patients reported the history of knee injury and surgery at the enrollment visit (OAI study protocol). Knee frequently bend count was recorded during follow-up visit, using 0–5 degree points to measure the different activities of knees.

### Radiographs protocol

All participants underwent standard fixed knee x-ray measurement at baseline and at each subsequent follow-up visits. The established reader reference standard protocol was employed to assess KLG on the radiographs, with ROA being defined as KLG ≥ 2 [18]. MRI scans of the target knee were performed using 3T MRI systems (Magnetom Trio, Siemens, Erlangen, Germany) across the four OAI clinical sites. MR images were captured using coronal T2-weighted turbo spin echo (TSE), as detailed in the OAI protocol [19]. MR images were evaluated at P-0, P-1 and BL. The MRI image database was subsequently transferred to a separate workstation and manually evaluated by a trained reader using the Mimics software version 19.0 [20] and assessed by trained readers to treatment without knowing participants’ group assignments. We used GE Optima CT 680 to collect CT images of a patient in our hospital, with a slice thickness of 0.625 mm. We also used MR PHILIPS Achieva 3.0T to collect magnetic resonance imaging (MRI) images of the subject, with a slice thickness of 1.0 mm. All MRIs were read sequentially, blinded to the time point, participant clinical data and grouping status.

### Magnetic resonance imaging assessment

**Fig. 1:**
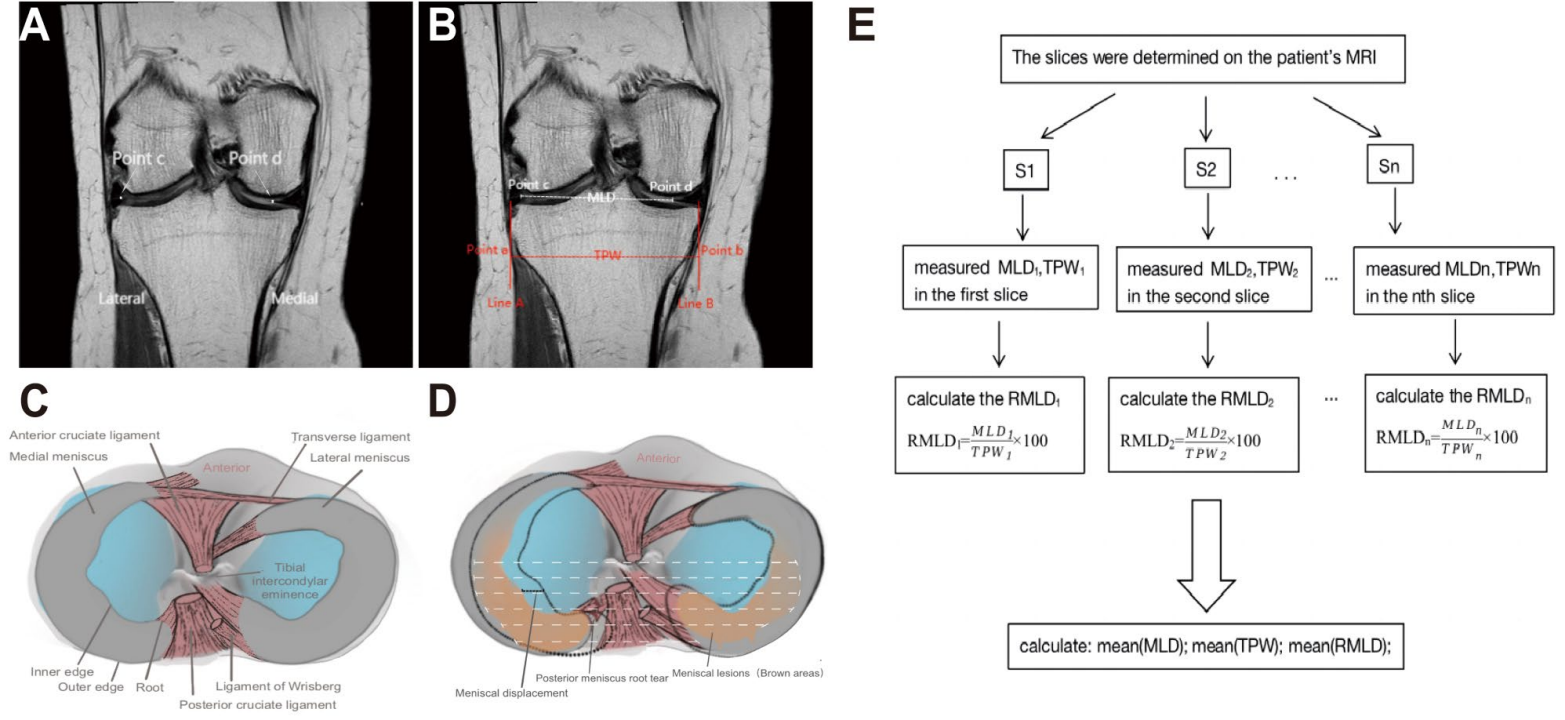
Meniscus parameters measurement and measurement flow chart. In Fig. 1A, B, Point c corresponds to the most lateral edge of a continuous lesion beginning from the inner edge of the lateral meniscus, while point d represents the most medial edge of a continuous lesion initiating from the inner edge of the medial meniscus. In Fig. 1B, Line B intersected the peripheral margin of the lateral tibial plateau and Line A intersected the inboard margin of the lateral tibial plateau; the distance between these two lines was defined as TPW. Figure 1C displays a normal knee joint model. Figure 1D presents a simplified lesion model, demonstrating various types of lesions potentially affecting the new meniscal parameters. MLD: Six parallel white dashed lines represent the medial to lateral meniscus lesion distance

In the evaluation process, coronal T2-weighted TSE MRI images were utilized to ascertain the mean distance between lesions on the medial and lateral menisci (MLD) and the width of the tibial plateau (TPW). These measurements were conducted on MRI slices ranging from the tibial eminence (initiating at the most elevated point observable on the MRI slice) to the posterior horn (terminating at the final slice of the meniscus in MRI). Such measurements were manually executed and quantitatively assessed (as depicted in Fig. 1). The Mean values of MLD and TPW across all measured slices from the tibial eminence to the posterior horn of the meniscus on MR Images were defined as Mean (MLD) and mean (TPW) respectively. The lesion’s distance of the medial meniscus to lateral meniscus (MLD) was defined as the distance from the innermost edge of a continuous lesion (taking the body as the reference axis) beginning from the inner edge of the medial meniscus (taking the knee as the reference axis) to the outermost edge of a continuous lesion (taking the body as the reference axis) initiating from the inner edge of the lateral meniscus (taking the knee as the reference axis) (Fig. 1A, B). In the absence of meniscal lesions, the MLD was defined as the distance from the medial edge of the medial meniscus to the line connecting the medial edge of the lateral meniscus (using the knee as the reference axis). MLD is the distance between the degeneration signal of the medial meniscus and the degeneration signal of the lateral meniscus (the width of coronal meniscus degeneration signal in MRI was measured completely). The relative percentage of the meniscal lesions distance (RMLD) was calculated as the MLD divided by the width of the tibial plateau, expressed as a percentage. The Mean(RMLD) represents the average value of the RMLD across all measured slices in the knee MRI.

Quantitative measurements of the morphological parameters for the medial-to-lateral meniscus, specifically Mean(MLD), Mean(TPW) and Mean(RMLD), were taken at three stages: P-0, P-1 and baseline in the knee joint. Figure 1C presents a model of a normal knee joint, illustrating the anatomical structure of a portion of the knee joint. Figure 1D displays a simplified lesion model, demonstrating various lesion types that may influence the newly defined meniscal parameters. The white dashed line approximately delineates the starting and ending points of our measurements and also defines the measurement layers. This line may also be interpreted as the distance from the medial-to-lateral meniscal lesions (MLD). The segmentation was limited to all slices ranging from the tibial intercondylar spine to the posterior aspect of the knee, which are typically the most representative sites of meniscal lesions [21]. Subsequently, we determined the mean lengths of MLD, TPW and RMLD across all slices extending from the intercondylar spine to the posterior of the knee joint (depicted as the average length of the white dashed lines in Fig. 1D), denoted as Mean(MLD), Mean(TPW), and Mean(RMLD) values, respectively.

Figure 1E provides a flowchart detailing the measurement process. S1, S2, … Sn denote consecutive measurement slices within the specified region of interest on the MRI, which extends from the tibial eminence (starting at the highest position displayed on the MRI slice) to the posterior horn (the final slice of the meniscus on the MRI) of the meniscus. Measurements for MLD_1_ and TPW_1_ were taken at the S1 slice, as detailed subsequently, while MLD_2_ and TPW_2_ were measured at the S2 slice using the same methodology. This procedure was repeated until all selected slices of interest were sequentially measured. The ratio of MLD to TPW (RMLD) was computed as a percentile for each MRI slice within the region of interest, with the outcome of the first slice labeled as RMLD_1_, the second slice as RMLD_2_, and so forth, until the final slice result, marked as RMLDn.

The methods employed to measure the MLD involved the identification of two specific points—point c and point d—on the MRI slice (Fig. 1A, B). Point c was defined as the most lateral edge of a continuous lesion starting from the inner margin of the lateral meniscus, utilizing the knee as the reference axis. Conversely, point d was defined as the most medial edge of a continuous lesion originating from the inner margin of the medial meniscus, again using the knee as the reference axis. In instances where no meniscal lesion signal was detected, points c and d corresponded to the inner margin of the lateral and medial meniscus, respectively (utilizing the knee as the reference axis).

The TPW measurement was accomplished by drawing two vertical lines (Line A and Line B) on the image (Fig. 1B). Line B intersected the peripheral margin of the lateral tibial plateau, while Line A intersected the inboard margin of the same plateau. The distance between these two lines defined the TPW. Importantly, any osteophytes suspected on the tibial plateau were excluded from the TPW measurement.

We then calculated the mean values of MLD, TPW, and RMLD (annotated respectively as “Mean(MLD),” “Mean(TPW),” and “Mean(RMLD)”). The computations for these means followed the formulas described below. (n: Number of slices measured within the region of interest; MLDn: Measurements of MLD on the nth slice MRI)

$${\text{Mean}}\left( {{\text{MLD}}} \right){\text{ = }}\frac{{{\text{ML}}{{\text{D}}_{\text{1}}}{\text{ + ML}}{{\text{D}}_{\text{2}}}{\text{ + \ldots \ldots + ML}}{{\text{D}}_{\text{n}}}}}{{\text{n}}}$$

$${\text{Mean}}\left( {{\text{TPW}}} \right){\text{ = }}\frac{{{\text{TP}}{{\text{W}}_{\text{1}}}{\text{ + TP}}{{\text{W}}_{\text{2}}}{\text{ + \ldots \ldots + TP}}{{\text{W}}_{\text{n}}}}}{{\text{n}}}$$

$${\text{Mean}}\left( {{\text{RMLD}}} \right){\text{ = }}\frac{{{\text{RML}}{{\text{D}}_{\text{1}}}{\text{ + RML}}{{\text{D}}_{\text{2}}}{\text{ + \ldots \ldots + RML}}{{\text{D}}_{\text{n}}}}}{{\text{n}}}$$

In addition, the relevant mechanism study data were processed according to the following methods for the imaging data of the healthy adult male collected in our hospital. The DICOM format CT and MRI images were imported into MIMICS v21.0 (Materialise, Leuven, Belgium). A three-dimensional model of the femur, tibia, and fibula was constructed using the CT images, while the three-dimensional models of the meniscus and cartilage were constructed using the MRI images. These models were then exported as STL files and imported into Geomagic Wrap software (version 2021, Geomagic Corporation, USA) for smoothing and surface generation, and subsequently exported as STP files. The STP files were further imported into SolidWorks 2023 software (Dassault Systemes, S.A., USA) for assembling the solid models. In Mimics, tears were created in the posterior horns of both menisci to simulate degenerated menisci. In SolidWorks software, the menisci were displaced laterally to create a bigger Mean(MLD) and a bigger Mean(RMLD) for the meniscus. Thus, four groups of three-dimensional models were obtained: normal state (control), meniscal extrusion (ME), meniscal degeneration (MD), and Meniscus with extrusion and degeneration (MED) (Fig. 4A, B, C, D). Finally, the assembled solid models from SolidWorks were imported into ANASYS 2022 software (ANASYS Corp, USA). According to previous studies, the material parameters for each part were set as follows (Table 1): the Young’s modulus of cortical bone was set to 16,800 MPa with a Poisson’s ratio of 0.3 [22]. The Young’s modulus of cancellous bone was set to 840 MPa with a Poisson’s ratio of 0.2 [23]. The Young’s modulus of cartilage was set to 12 MPa with a Poisson’s ratio of 0.45. The Young’s modulus of the meniscus was set to 80 MPa with a Poisson’s ratio of 0.3 [24]. In this study, it was assumed that there was sliding-limited frictionless contact between the articular cartilage of the femur and tibia, and between the articular cartilage of the tibia and meniscus. The friction coefficient between the upper part of the meniscus and the articular cartilage of the femur was set to 0.2. A vertical load was applied to the cross-section of the femur in each three-dimensional model, with a magnitude of weight (kg) × 10 (m/s²) / 2. At the same time, the distal ends of the tibia and fibula in each three-dimensional model were fixed.

**Table 1:** Material properties of each component

Item	Young’s modulus	Poisson’s ratio
**Cortical bone**	16,800	0.3
**Cancellous bone**	840	0.2
**Meniscus**	80	0.3
**Cartilage**	12	0.45

To explain the effect of parameters on tibial cartilage load. Nevertheless, it is difficult to study the stress of joints in subjects, because no sensor can be inserted into the human body without causing harm. Therefore, we used finite element analysis to explore the effect of meniscal parameters alteration on tibial cartilage mechanics. In the study of mechanical mechanisms, it was assumed that there was sliding-limited frictionless contact between the articular cartilage of the femur and tibia, and between the articular cartilage of the tibia and meniscus [25]. The friction coefficient between the upper part of the meniscus and the articular cartilage of the femur was set to 0.2 [22]. A vertical load was applied to the cross-section of the femur in each three-dimensional model, with a magnitude of weight (kg) × 10 (m/s²) / 2 [26]. At the same time, the distal ends of the tibia and fibula in each three-dimensional model were fixed. Finite element analysis model, the von Mises stress distribution of the femoral cartilage and their maximum von Mises stress values were calculated using the software for each of the four states: normal state (control), meniscal extrusion (ME), meniscal degeneration (MD), and Meniscus with extrusion and degeneration (MED).

### Statistical analysis

A reader measured the novel parameters in all MRI images. For assessing reliability, these parameters of meniscus were measured by the same reader twice with a one-month gap for intra-class reliability (intra-class correlation coefficients, ICCs) and by two readers independently for inter-class reliability (inter-class correlation coefficients) [27] in a set of 51 MRI images chosen at random. The normality of the data was tested by Shapiro-Wilk test. Categorical data were presented as the number (percent), and continuous data were expressed as the means and standard deviations or as the medians and interquartile ranges as appropriate. Student’s T- test, and Chi-Squared tests was used to compare statistic difference between case and control groups. Conditional logistic regression analyses were applied to determine the risk of incident ROA in participants, both before and after adjustments for covariates including BMI, previous knee surgery, previous knee injury and frequently bend count at baseline. Sensitivity analysis was performed for P-0, P-1 and baseline. Models run at three time points: P-0, P-1 and baseline. All statistical analyses were measured and evaluated on a subject basis using a double-blind method using R-Studio software (x64 4.2.2), with *P*-values less than 0.05(double-tailed) considered statistically significant.

## Results

### Characteristics of the participants

**Fig. 2:**
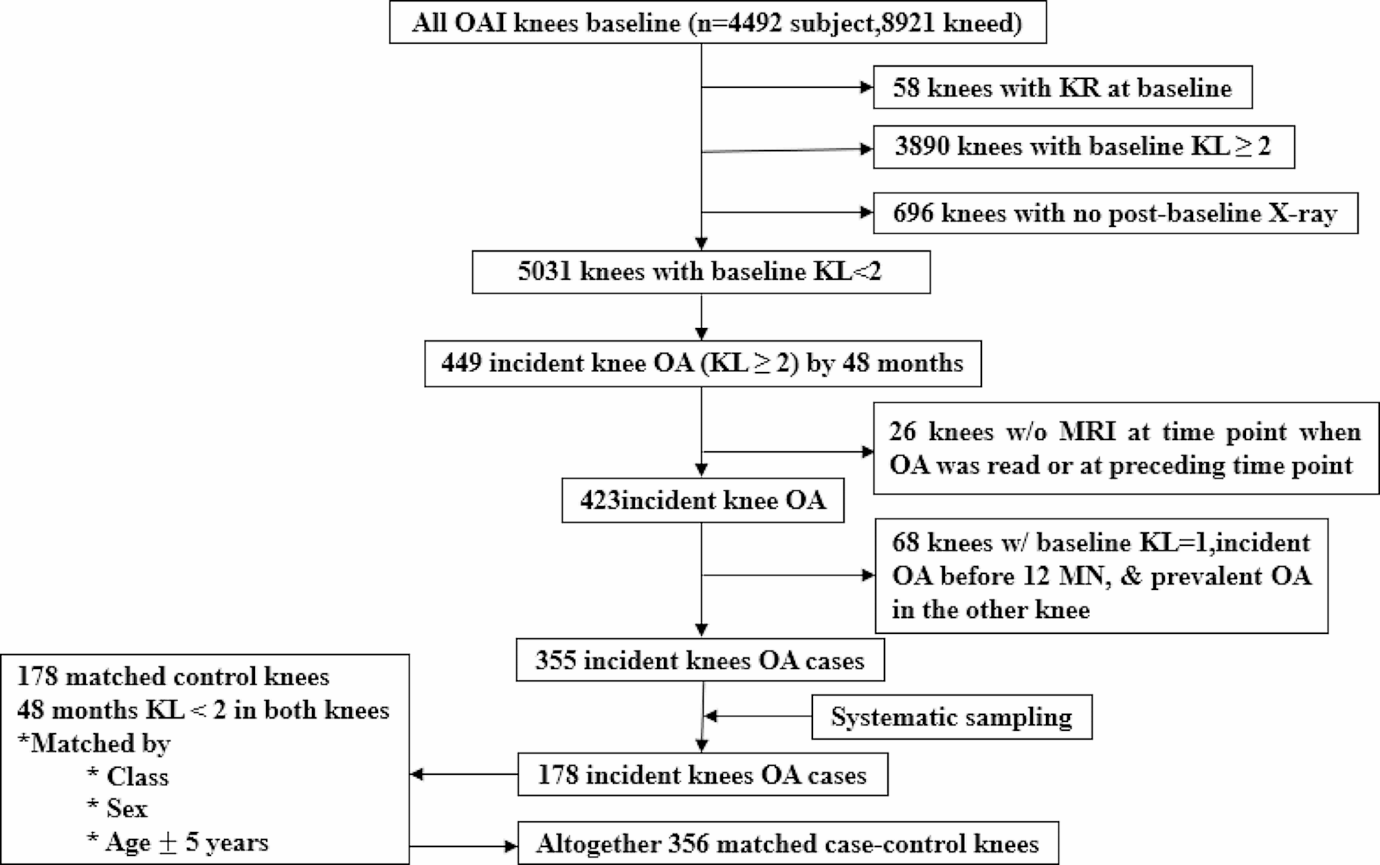
Flowchart illustrating patient selection process

The baseline characteristics of the participants are shown in Table 2. A flowchart illustrating the full patient selection process can be found in Fig. 2. The study encompassed a total of 356 knees from 354 participants who had an average age of 60.22 years (SD: 8.53). Participants were predominantly female, accounting for 66.5% in the incident ROA group and 66.9% in the group with no incident ROA. The majority were overweight with a mean BMI of 28.64 in the incident ROA group, and 27.68 in the group with no incident ROA. The age distributions were similar between the case and control groups (mean age: 60.25 years ± 8.65 SD vs. 60.19 years ± 8.46 SD, *p* = 0.872). The two groups were also comparable regarding age, sex, race, baseline radiographic features, and baseline injury. However, the case group exhibited higher BMI levels, a finding statistically significant (*p* < 0.05). In comparison to the control group, the case group had a higher history of injury (39.9% vs. 19.9%, *p* < 0.001) and surgery (15.7% vs. 7.3%, *p* = 0.023). Differences were also noted in the baseline frequency of bending between the case and control groups.

**Table 2:** Demographics of participants with incident ROA vs. those without incident ROA participants

characteristics	Per person			
		Incident ROA(*n* = 176)	No incident ROA(*n* = 178)	*P*-value
Age, years	Mean(SD)	60.25(8.65)	60.19(8.46)	0.872
Gender, %	Female	117(66.5)	119(66.9)	1.000
	Male	59 (33.5)	59(33.1)	
Race, %	White	144(81.8)	149(83.7)	0.735
	Non-White	32(18.2)	29(16.3)	
BMI, kg/m^2^	Mean(SD)	28.64(4.61)	27.68(4.44)	0.032
	**Per knee**			
		**Incident ROA(** *n* ** = 178)**	**No incident ROA(** *n* ** = 178)**	***P-value***
KLG, %	0	67(37.6)	67(37.6)	1.000
	1	111(62.4)	111(62.4)	
History of knee injury, %	No	107(60.1)	143(80.3)	< 0.001
	Yes	71(39.9)	35(19.7)	
BL injury, %	No	132(74.2)	146(82.0)	0.100
	Yes	46(25.8)	32(18.0)	
History of surgery, %	No	150(84.3)	165(92.7)	0.023
	Yes	28(15.7)	13(7.3)	
Baseline frequent bend count, %	No	11(6.2)	23(12.9)	0.031
	1,2 or 3	130(73.0)	131(73.6)	
	4 or 5	37(20.8)	24(13.5)	

Data presented as mean (S.D.) or n (%).

### Reliability

The intra-class and inter-class correlation coefficients for measurements of the novel parameters are presented in Table 3. Both the intra-class and inter-class reliabilities of all new parameter measurements were excellent (≥ 0.91).

**Table 3:** Intra-class and inter-class correlation coefficients

	Intra-class correlation (95%CI)	inter-class correlation (95%CI)
**Mean(MLD)**	0.95(0.92, 0.97)	0.95(0.91, 0.97)
**Mean(TPW)**	0.99(0.99, 1.00)	0.99(0.98, 1.00)
**Mean(RMLD)**	0.93(0.89, 0.96)	0.91(0.85, 0.95)

### Comparison of the morphological parameters between incident ROA cases and controls

**Fig. 3:**
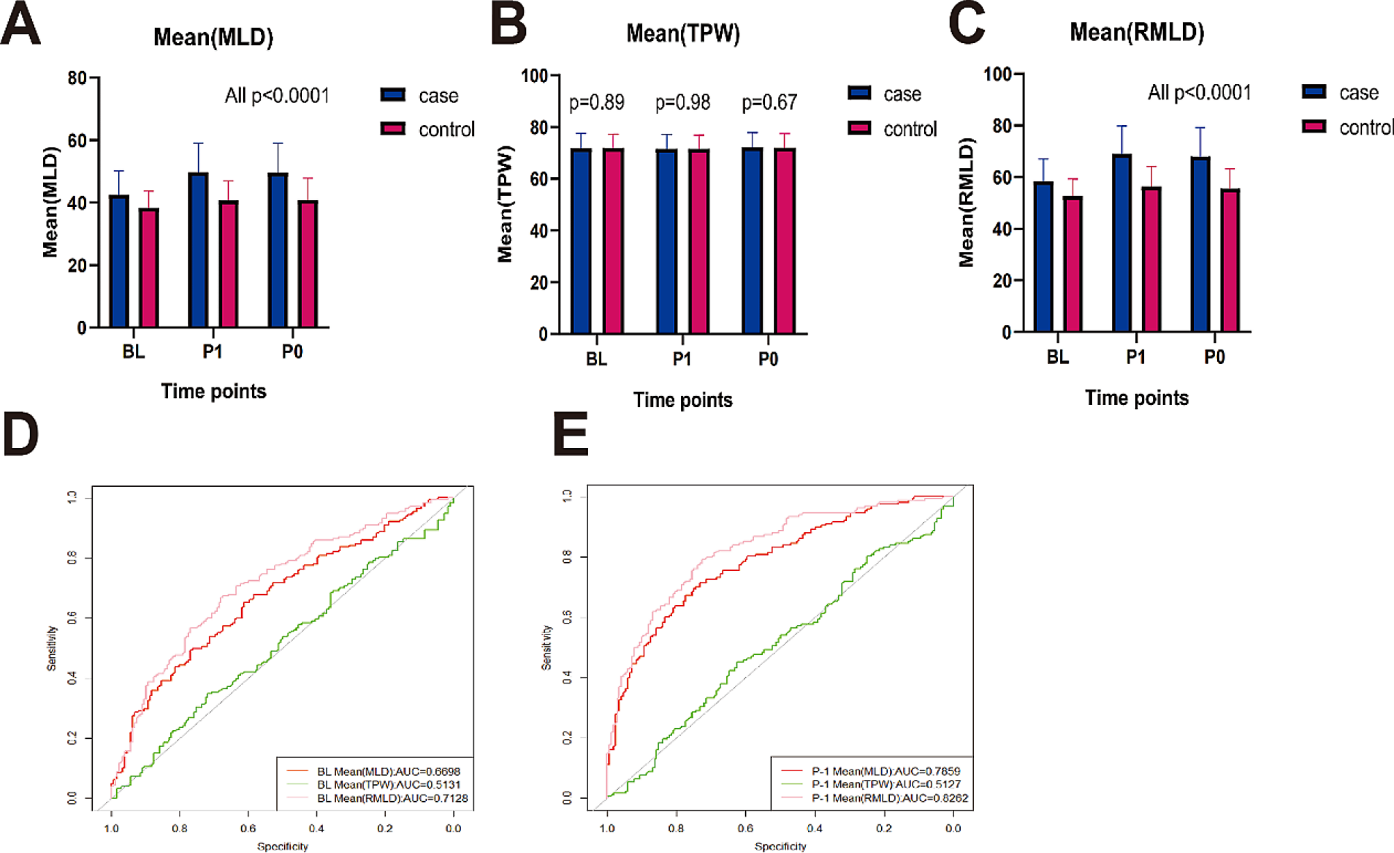
Statistical analysis of meniscus lesion parameters in case group and control group. Panel **(A)**,** (C)** displays statistically significant differences in Mean(MLD) and Mean(RMLD) between two groups at BL, P-1and P-0, respectively. Panel **(B)** reveals no significant differences in Mean(TPW) between the groups at BL, P-1, and P-0. **(D)** shows the predictive effect values of parameters at baseline for predicting ROA occurrence. **(E)** shows the predictive effect values of parameters at P-1 for predicting ROA occurrence

Figure 3A, B and C showed a comparison of measurements of novel meniscus parameters between the case and control groups. Table 4 presents the mean and standard deviation of these meniscus parameters at BL, P-1, and P-0. Compared to the control group, the case group exhibited significantly higher Mean (MLD) and Mean (RMLD) all time points (BL, P-1 and P-0), and the differences were statistically significant. However, no significant difference were found in the Mean (TPW) between the two groups at BL, P-1, and P-0, with respective *p* values of 0.892, 0.984 and 0.665.

**Table 4:** Means and SD for morphological parameters of the meniscus in incident vs. no incident ROA knees

			Incident ROA Mean ± SD	No incident ROA Mean ± SD	*P*-value
**BL**	**Mean(MLD)**, mm		42.56(7.70)	38.14(5.51)	< 0.001
	**Mean(TPW)**, mm		71.77(5.76)	71.85(5.37)	= 0.892
	**Mean(RMLD)**		58.61(8.52)	52.52(6.53)	< 0.001
**P-1**	**Mean(MLD)**, mm		49.73(9.39)	40.70(6.24)	< 0.001
	**Mean(TPW)**, mm		71.51(5.64)	71.52(5.37)	= 0.984
	**Mean(RMLD)**		68.95(10.94)	56.35(7.85)	< 0.001
**P-0**	**Mean(MLD)**, mm		49.57(9.52)	40.78(7.05)	< 0.001
	**Mean(TPW)**, mm		72.24(5.65)	71.98(5.47)	= 0.665
	**Mean(RMLD)**		67.93(11.40)	55.64(7.69)	< 0.001

### Associations of the morphological parameters changes of meniscus with ROA risk

During the baseline period, P-1 and P-0, the associations between knee measures and the incidence of ROA are shown in Table 5. During the baseline period, unadjusted analyses demonstrate that the baseline measurements of Mean(MLD) and Mean(RMLD) bear significant associations with the occurrence of ROA over four years. These correlations remain stable even after adjusting for BMI, previous knee surgery, previous knee injury and frequently bend count [Adjusted OR, 95%CI: 1.11(1.07 to 1.16) and 1.13(1.09 to 1.17), respectively]. However, the baseline Mean(TPW) did not show a significant association with incident ROA before and after adjustment for these factors [Adjusted OR, 95%CI: 0.98(0.94 to 1.02)].

**Table 5:** Associations between novel parameters for knees measures and incident ROA at baseline, P-1 and P-0

		Univariable		Multivariable^a^
		OR(95%CI)		OR(95%CI)
**BL**	**Mean(MLD)**	**1.11(1.06,1.15)**		**1.11(1.07,1.16)**
	**Mean(RMLD)**	**1.11(1.08**,**1.15)**		**1.13(1.09**,**1.17)**
	**Mean(TPW)**	1.00(0.96,1.04)		0.98(0.94,1.02)
**P-1**	**Mean(MLD)**	**1.16(1.12**,**1.20)**		**1.15(1.11**,**1.20)**
	**Mean(RMLD)**	**1.15(1.11**,**1.18)**		**1.14(1.11**,**1.18)**
	**Mean(TPW)**	1.00(0.96,1.04)		0.98(0.94,1.02)
**P-0**	**Mean(MLD)**	**1.13(1.10**,**1.17)**		**1.13(1.09**,**1.17)**
	**Mean(RMLD)**	**1.14(1.10**,**1.17)**		**1.13(1.10**,**1.17)**
	**Mean(TPW)**	1.01(0.97,1.05)		0.99(0.95,1.04)

^a^Adjusted for BMI, previous knee surgery, previous knee injury and frequently bend count. BL: baseline; P-1: one year prior the occurrence of ROA;P-0: Time to visit when ROA was found on the radiographs. Significant associations are shown in bold (*p* < 0.05).

In the P-1 period, both Mean(MLD) and Mean(RMLD) were significantly and positively associated with the incidence of ROA after 1 year before and after adjustment for BMI, previous knee surgery, previous knee injury and frequently bend count [Adjusted OR(95% CI): 1.15 (1.11 to 1.20) ; 1.14 (1.11 to 1.18)]. Interestingly, these odds ratios were marginally larger than those at the baseline. The P-1 Mean(TPW) did not exhibit any significant association with the onset of ROA after 1 year, even before and after adjustment for covariates.

When ROA was found on the radiographs, both Mean(MLD) and Mean(RMLD) were significantly positively correlated with ROA occurrence before and after adjustment for covariates. This relationship was statistically significant [Adjusted OR (95% CI): 1.13(1.09,1.17); 1.13 (1.10 to 1.17)]. Conversely, Mean(TPW) at P-0 did not exhibit a significant association with incident ROA before and after adjustment for covariates.

Two receiver operating characteristic (ROC) curves were constructed to evaluate the area under the curve (AUC) and thus, ascertain the predictive value of the parameters at BL and P-1 (Fig. 3D, E). The ROC curves indicated that both the Mean(MLD) and Mean(RMLD) at BL and P-1 were statistically significant in predicting incident ROA [BL Mean(MLD) AUC: 0.6698; *P* < 0.001; 95% CI, 0.614–0.726; BL Mean(RMLD) AUC: 0.7128; *P* < 0.001; 95% CI, 0.660–0.766; P-1 Mean(MLD) AUC: 0.7859; *P* < 0.001; 95% CI, 0.738–0.834; P-1 Mean(RMLD) AUC: 0.8262; *P* < 0.001; 95% CI, 0.783–0.870]. In contrast, the Mean(TPW) at both BL and P-1 did not exhibit significant predictive value for incident ROA, with AUC values of 0.5131 [95% CI, 0.453–0.573] and 0.5127 [95% CI, 0.451–0.575], respectively.

### Relationship between the parameters changes of meniscus and tibial cartilage stress

**Fig. 4:**
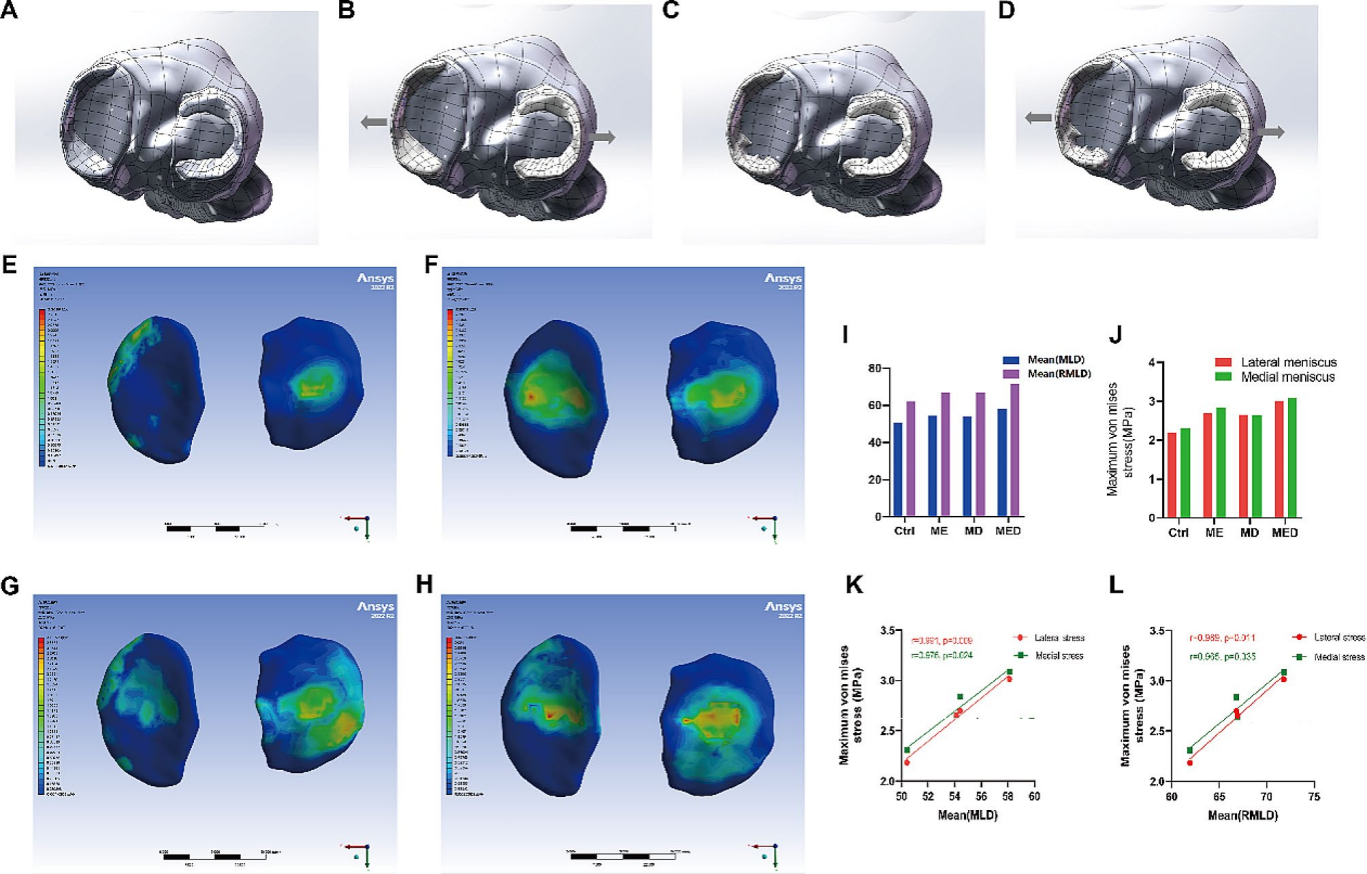
Establishment of four meniscus models of knee joint and von Mises stress distribution of tibial cartilage in different model states. **A**: the normal knee joint model; **B**: meniscus displacement 2 mm model; **C**: meniscus degeneration model; **D**: meniscus degeneration with meniscus extrusion model. The von Mises stress distribution of the medial and lateral tibial cartilage in control (**E)**, group ME **(F)**, group MD **(G)** and group MED **(H)**. Figures I and J show the measurement of Mean(MLD) and Mean(RMLD) parameters and the analysis of the maximum von Mises stress value of meniscus models of group ME, MD MED and control. K and L show the correlation of meniscus parameters with the maximum von Mises stress of the medial and lateral tibial cartilages

The von Mises stress distribution of the tibial cartilage reflects the load magnitude on the tibial cartilage under the four different Mean(MLD) simulated conditions (the normal knee joint model; meniscus extrusion 2 mm model; meniscus degeneration model; meniscus degeneration with meniscus extrusion model). In the normal condition (Mean(MLD) and Mean(RMLD) are the smallest parameters of the four groups), both sides of the tibial cartilage experience lower stress (Fig. 4E). In the case of meniscal extrusion (Mean(MLD) and Mean(RMLD) are increased), the stress on both sides of the tibial cartilage significantly increases compared to the normal state, and the areas with increased stress are primarily located in the central region not covered by the meniscus (Fig. 4F). After meniscal degeneration (Mean(MLD) and Mean(RMLD) increased equally compared with meniscal extrusion group), the stress on both sides of the tibial cartilage also increases to a certain extent compared to the normal state, and the areas with increased stress are mainly located in the peripheral region that was originally covered by the meniscus before degeneration (Fig. 4G). In the case of Meniscus with extrusion and degeneration (Mean(MLD) and Mean(RMLD) are the largest of the four groups), the stress on both sides of the tibial cartilage is highest among these four conditions, with increased stress observed in both the central and peripheral regions (Fig. 4H). Figure 4I, J; Table 6 presents the measured parameters and the maximum von Mises stress values of each tibial cartilage in the four groups of three-dimensional models. Comparing the maximum von Mises stress of the tibial cartilage in the four groups, meniscal extrusion with degeneration (MED) group with maximum Mean(MLD) and Mean(RMLD) have significantly higher tibial cartilage stress than meniscal compression (ME) or degeneration (MD) group, both of which are significantly higher than normal (control) group with minimum Mean (MLD) and Mean (RMLD). Figure 4K and L show that Mean(MLD) and Mean(RMLD) are significantly positively correlated with maximum von Mises stress regardless of the type of meniscus disease (*r* = 0.991, 0.976, 0.989, and 0.965, respectively). Respectively).

**Table 6:** The measured parameters and the maximum Von Mises stress values of each tibial cartilage in the four groups

Item	Control	ME	MD	MED
**Mean(MLD)**	50.41	54.41	54.12	58.12
**Mean(RMLD)**	61.92	66.80	66.91	71.79
**Maximum von mises stress of lateral meniscus**,** MPa**	2.1839	2.6997	2.6557	3.0130
**Maximum von mises stress of medial meniscus**,** MPa**	2.3086	2.8392	2.6474	3.0866

ME: meniscal extrusion; MD: Meniscus degeneration; MED: meniscal displacement with degeneration.

## Discussion

Over the four-year follow-up period, Mean(MLD) and Mean(RMLD) values obtained at baseline and P-1 robustly predicted incident ROA. Our findings illustrate that, aside from Mean(TPW), all baseline parameter measurements, both before and after covariate adjustment, exhibited significant associations with knee OA development within a 4-year timeframe. Apart from Mean(TPW), all parameters gauged at P-1 anticipated the onset of ROA after 12 months and at P-0, maintaining a significant positive correlation with the onset of ROA before and after covariation-adjustment. Further, the novel quantitative assessment approach demonstrated excellent intra-class and inter-class reliability. Finite element analysis shows that Mean(MLD) and Mean(RMLD) have significant positive correlation with von Mises stress. These findings suggest that our quantitative measurements of the medial-to-lateral meniscal parameter alterations possess predictive validity.

Typically, meniscus alterations have been evaluated using semi-quantitative approaches such as WORMS and BLOKS. The Whole-Organ Magnetic Resonance Imaging Score (WORMS) scores of ≥ 2 are considered as meniscus tears, while a score of meniscal extrusion grade ≥ 1 indicates meniscus extrusion. BLOKS employs similar thresholds as WORMS, yet it categorizes meniscus tears on a scale of 0 to 6 and meniscus extrusion on a scale of 0 to 3, in contrast to the WORMS scale of 0 to 5 and 0 to 2, respectively [28, 29]. In recent years, some researchers have proposed quantitative methodologies for meniscus evaluation, which encompass parameters such as meniscal volume, meniscal width, meniscus extrusion and meniscus height, and define an absolute meniscus extrusion distance ≥ 3 mm as pathological meniscus extrusion [30]. However, several previous studies have suggested that MRI-assessed meniscus extrusion does not correlate with the risk of ROA development. For instance, Sharma et al. conducted a 24-month cohort study on osteoarthritis, and found no association between baseline semi-quantitative scoring of medial meniscus extrusion and cartilage volume changes [4]. Similarly, Roemer et al. conducted a nested case-control study over a period of 4 years, concluding that meniscus extrusion did not predict ROA [31]. Intriguingly, our current findings may have higher research value, and the parameters of meniscus lesions might offer more precise prediction for ROA onset (AUC 0.6698 [BL Mean(MLD)]; AUC 0.7128 [BL Mean(RMLD]; AUC 0.7859 [P-1 Mean(MLD)]; AUC 0.8262 [P-1 Mean(RMLD]), corroborating our prior hypothesis. Nevertheless, the AUC of parameters at the BL stage was smaller in comparison to those at the P-1 stage. This may be attributed to non-uniform changes in the parameters during various ROA periods, with significant alterations occurring during the near-onset period. By contrast, we can think that the combined indicators of these factors related to OA progression may have better predictive effect.

Consistent with the investigations conducted by Sharma et al. [4] and Roemer et al. [31], previous studies predominantly examined individual aspects of meniscus lesions, resulting in the potential loss of integrative insights [16, 32]. These research efforts typically concentrated on the independent alterations of the medial or lateral meniscus, which could potentially explain the non-significant findings. Our study, therefore, adopted a more comprehensive approach by focusing on the combined parameters of both the medial and lateral meniscus. Building upon the foundations of these preceding studies, we devised a set of novel composite indices for the meniscus. These indices were not only described in detail but their predictive validity was also thoroughly corroborated through this research. The quantitative method we employed demonstrated repeatability and effectiveness in measuring meniscal alterations, subsequently predicting the incidence of ROA accurately. Findings from our study revealed a significant association between both Mean(MLD) and Mean(RMLD) with the development and incidence of ROA, further affirming the predictive validity of the method at baseline and P-1.

The etiology of meniscus injury is multifaceted and its association with OA has been investigated extensively; however, the exact mechanism remains elusive. It has been proposed that meniscus maceration and degeneration might act as early indicators of ROA, as such degenerative changes can manifest as abnormal signals on MRI [33, 34]. These atypical signals, arising from knee degeneration changes, form an integral part of our measurement parameters. Traumatic meniscus tears, instigating mechanical alterations within the meniscus, may contribute to the onset of knee ROA. As ROA advances, the degradation and consequent weakening of the meniscus structure can potentially augment the incidence of tears, thereby accelerating the progression of knee ROA. Such tear signals are predominantly observed in the posterior corner of the meniscus, and may directly induce changes in our parameters [7, 35]. An additional mechanism could be the reduction in the elasticity and resilience of the meniscus during joint disintegration, leading to meniscal dislocation. This dislocation, over time, may cause a gradual increase in the medial-to-lateral meniscus lesions distance, subsequently leading to an increased MLD [36]. Furthermore, poor repair of the ligament of Wrisberg post-injury may heighten the risk of lateral meniscus displacement over time, possibly enlarging meniscal parameter measurements, akin to those seen with meniscus dislocation [37]. In addition, anterior cruciate ligament injuries have been identified as potential precursors for post-traumatic OA [38]. As previously mentioned, the predictive validity of utilizing meniscus extrusion measurements for forecasting ROA development can be contentious; however, research from several scholars has established a correlation between meniscus extrusion and the escalation of knee ROA, knee discomfort, total knee arthroplasty in knee arthritis patients, and structural advancement [7, 39]. It has been observed that a more severe meniscus extrusion results in an increased distance between the medial and lateral menisci, thus expanding the metric MLD. This accumulation of OA risk factors potentially enhances the predictive accuracy of the current measurements. Currently, we can think that this newly introduced parameter is a comprehensive index that represents these risk factors. Finally, we used the finite element analysis method to explore the stress on tibial cartilage under different meniscus lesions. We found that regardless of the disease, when a certain force is applied to the upper end of the femur, the tibial stress point and the magnitude of the force will change with the increase of Mean(MLD) or Mean(RMLD), which may be the reason for predicting the effective parameters of OA.

Meanwhile, from this series of studies, the role of meniscus in OA should not be underestimated. With the development of regenerative medicine and 3D printing technology, the production of human tissues and organs for surgical treatment and transplantation has given researchers hope that bionic meniscus or cartilage printed by meniscus tissue analogues or hydrogels can be used for human transplantation materials and scientific research materials [40]. In addition, the knee joint structure was 3D printed with materials with similar parameters such as meniscus cartilage, bone and ligament, and then the personalized 3D knee joint model was reconstructed in vitro. In vitro model, mechanical sensors were used to measure the force variation of knee joint under different meniscus parameters, and the influence of meniscus parameter variation on knee joint force was simulated. Finally, the meniscus parameters that best fit the physiological characteristics of the knee joint are found, which may help guide the clinical surgical treatment of OA patients and the development of artificial knee prostheses. This will be the focus of further research work.

Although we validated the validity of composite parameters in predicting incident ROA in a nested case-control study, we must emphasize several limitations in the current study. Firstly, the measurement of these meniscal parameters is a manual process, making it laborious and time-intensive when contrasted with the automatic measurement of artificial intelligence. Yet, manual segmentation does not depend on high-end hardware equipment [41]. Secondly, we acknowledge that our epidemiological study did not include pathological examinations, hence the pathological alterations coinciding with meniscal parameter changes remain undefined. Thirdly, the study focused solely on lesions originating from the meniscus’s inner edge, disregarding the potential contribution of lesions from the meniscus’s outer edge to ROA. Fourthly, the structural clinical validity of the meniscal parameters needs comprehensive investigation, a subject we intend to explore in future research. Finally, it should be noted that our case group demonstrated higher incidences of obesity, surgery, baseline frequent bend count, and injury compared to the control group, potentially impacting our results. Nevertheless, these variables were integrated as potential confounders into the analytical models and therefore our findings should not be greatly affected by these factors.

## Conclusions

In summary, our study demonstrated a statistically significant elevation in both Mean (MLD) and Mean (RMLD) at baseline, P-1 and P-0 in the case group as compared to the control group. Nevertheless, the Mean(TPW) did not display a significant difference between the two cohorts. The enhancement of these innovative meniscal parameters echoes prior research, further reinforcing their utility in forecasting the incidence of ROA. These results emphasize the crucial role of the meniscus in the pathogenesis of ROA. The quantitative measurement of meniscus is sensitive to meniscus lesions and can be used as an ideal endpoint for intervention. The early identification and intervention of meniscal abnormalities might serve to impede the progression of ROA, highlighting the potential for preventive strategies.

### Electronic supplementary material

Below is the link to the electronic supplementary material.

Supplementary Material 1

Supplementary Material 2

## Data Availability

The datasets utilized and/or analyzed during the current study are accessible from the corresponding author upon reasonable request.

## Abbreviations

ROA: Radiographic knee osteoarthritis
OAI: Osteoarthritis Initiative
KLG: Kellgren Lawrence Grade
BL: baseline
TSE: Turbo spin echo
P-0: Time to visit when ROA was found on the radiographs
P-1: One year prior to P-0
OA: Osteoarthritis
WORMS: Whole Organ Magnetic Resonance Imaging Score
BLOKS: Boston Leeds Osteoarthritis Knee Score
MOAKS: MRI Osteoarthritis Knee Score
MLD: The medial and lateral meniscal lesions distance
Mean(MLD): The mean of the medial and lateral meniscal lesions distance
TPW: Tibial plateau width
Mean(TPW): The mean of tibial plateau width
RMLD: Relative percentage of the medial and lateral meniscal lesions distance
Mean(RMLD): the mean of the relative percentage of the medial and lateral meniscal lesions distance
BMI: Body mass index
TKR: total knee replacement
MRI: Magnetic resonance imaging
ICCs: intraclass correlation coefficients
SD: Standard deviation
OR: Odds ratio
CI: Confidence interval, ME: meniscal extrusion
MD: Meniscus degeneration
MED: meniscal displacement with degeneration
