# Supplementary material for: Predicting incident radiographic knee osteoarthritis through quantitative meniscal lesion parameters: data from the osteoarthritis initiative
Source: BMC Musculoskelet Disord. 2024 Aug 6;25:626. doi: 10.1186/s12891-024-07706-5 (PMC11304704; doi:10.1186/s12891-024-07706-5)
Supplement: Supplementary file 1 — Supplementary Material 1 [file 12891_2024_7706_MOESM1_ESM.docx]

**Table1 Material properties of each component**

| **Item** | **Young's modulus** | **Poisson's ratio** |
| --- | --- | --- |
| **Cortical bone** | 16800 | 0.3 |
| **Cancellous bone** | 840 | 0.2 |
| **Meniscus** | 80 | 0.3 |
| **Cartilage** | 12 | 0.45 |
