# Supplementary material for: Predicting incident radiographic knee osteoarthritis through quantitative meniscal lesion parameters: data from the osteoarthritis initiative
Source: BMC Musculoskelet Disord. 2024 Aug 6;25:626. doi: 10.1186/s12891-024-07706-5 (PMC11304704; doi:10.1186/s12891-024-07706-5)
Supplement: Supplementary file 2 — Supplementary Material 2 [file 12891_2024_7706_MOESM2_ESM.docx]

**Table 3 Intra-class and inter-class correlation coefficients**

|  | **Intra-class correlation (95%CI)** | **inter-class correlation (95%CI)** |
| --- | --- | --- |
| **Mean(MLD)** | 0.95(0.92, 0.97) | 0.95(0.91, 0.97) |
| **Mean(TPW)** | 0.99(0.99, 1.00) | 0.99(0.98, 1.00) |
| **Mean(RMLD)** | 0.93(0.89, 0.96) | 0.91(0.85, 0.95) |
